# Challenges and opportunities of developing bioinformatics platforms in Africa: the case of BurkinaBioinfo at Joseph Ki-Zerbo University, Burkina Faso

**DOI:** 10.1093/bib/bbaf040

**Published:** 2025-02-03

**Authors:** Ezechiel B Tibiri, Palwende R Boua, Issiaka Soulama, Christine Dubreuil-Tranchant, Ndomassi Tando, Charlotte Tollenaere, Christophe Brugidou, Romaric K Nanema, Fidèle Tiendrebeogo

**Affiliations:** Laboratoire de Virologie et de Biotechnologies Végétales, Institut de l’Environnement et de Recherches Agricoles (LVBV/INERA), Centre National de la Recherche Scientifique et Technologique (CNRST), 01BP476 Ouaga 01, Ouagadougou, Burkina Faso; Clinical Research Unit of Nanoro, Institut de Recherche en Sciences de la Santé, CNRST, 42 Avenue Kumda-Yonré, 218 Ouaga CMS 11, Nanoro, Burkina Faso; MRC Unit The Gambia, London School of Hygiene and Tropical Medicine, Atlantic Boulevard, Fajara, PO Box 273, Banjul, the Gambia; Sydney Brenner Institute for Molecular Biosciences (SBIMB), University of the Witwatersrand, The Mount, First Floor, Office 109, 9 Jubilee Road, Parktown, Johannesburg, South Africa; Institut de Recherche en Sciences de la Santé, Biomedical and Public Health Department, CNRST, Rue 29. 13 Wemtenga 03 BP 7047, Ouagadougou, Burkina Faso; DIADE, University of Montpellier, CIRAD, IRD, 911 Avenue Agropolis, Montpellier, Cedex 5 34934, France French Institute of Bioinformatics (IFB)—South Green Bioinformatics Platform, Bioversity, CIRAD, INRAE, IRD, F-34398 Montpellier, France; DIADE, University of Montpellier, CIRAD, IRD, 911 Avenue Agropolis, Montpellier, Cedex 5 34934, France French Institute of Bioinformatics (IFB)—South Green Bioinformatics Platform, Bioversity, CIRAD, INRAE, IRD, F-34398 Montpellier, France; PHIM, Plant Health Institute of Montpellier, Univ. of Montpellier, IRD, CIRAD, INRAE, Institut Agro, 911 Av. Agropolis, 34394 Montpellier, France; PHIM, Plant Health Institute of Montpellier, Univ. of Montpellier, IRD, CIRAD, INRAE, Institut Agro, 911 Av. Agropolis, 34394 Montpellier, France; Genetic and Plant Breeding Team (EGAP), Biosciences Laboratory, Doctoral School of Science and Technology, Joseph KI-ZERBO University, avenue Pr Yembila Abdoulaye Toguyeni, 03 BP 7021, Burkina Faso; Laboratoire de Virologie et de Biotechnologies Végétales, Institut de l’Environnement et de Recherches Agricoles (LVBV/INERA), Centre National de la Recherche Scientifique et Technologique (CNRST), 01BP476 Ouaga 01, Ouagadougou, Burkina Faso; Central and West African Virus Epidemiology (WAVE), Pôle scientifique et d’innovation de Bingerville, Université Félix Houphouët-Boigny (UFHB), Bingerville BPV 34 Abidjan, Côte d’Ivoire

**Keywords:** bioinformatics, BurkinaBioinfo, challenges, training

## Abstract

Bioinformatics, an interdisciplinary field combining biology and computer science, enables meaningful information to be extracted from complex biological data. The exponential growth of biological data, driven by high-throughput omics technologies and advanced sequencing methods, requires robust computational resources. Worldwide, bioinformatics skills and computational clusters are essential for managing and analysing large-scale biological datasets across health, agriculture, and environmental science, which are crucial for the African continent. In Burkina Faso, the establishment of bioinformatics infrastructure has been a gradual process. Initial training initiatives between 2015-2016, including bioinformatics courses and the establishment of the BurkinaBioinfo (BBi) platform, marked significant progress. Over 250 scientists have been trained at diverse levels in bioinformatics, 105 user accounts have been created for high-performance computing access. Operational since 2019, this platform has significantly facilitated training programs for scientists and system administrators in west Africa, covering data production, introductory bioinformatics, phylogenetic analysis, and metagenomics. Financial and technical support from various sources has facilitated the rapid development of the platform to meet the growing need for bioinformatics analysis, particularly in conjunction with local 'wet labs'. Establishing a bioinformatics cluster in Burkina Faso involved identifying the needs of researchers, selecting appropriate hardware and installing the necessary bioinformatics tools. At present, the main challenges for the BBi platform include ongoing staff training in bioinformatics skills and high-level IT infrastructure management in the face of growing infrastructure demands. Despite these challenges, the establishment of a bioinformatics platform in Burkina Faso offers significant opportunities for scientific research and economic development in the country.

## Why developing bioinformatics platforms in west Africa?

Bioinformatics has become increasingly important in recent years, particularly for analysing the flood of biological data following the explosion of high-throughput omics technologies and advances in sequencing techniques [1]. in many areas of life sciences research, including genomics, proteomics and metabolomics [2]. For example, bioinformatics can help identify potential targets for drug development, understand disease mechanisms and develop personalised medicine [3]. In agriculture, bioinformatics can be used to facilitate the development of new crop varieties with increased yield and resistance to pests and diseases [4]. Bioinformatics also has applications in environmental and conservation research, such as understanding biodiversity and ecosystem dynamics [4].

With advances in high-throughput technologies such as next-generation sequencing (Illumina, PacBio, Nanopore) and microarray analysis, the amount of biological data generated has increased exponentially in recent years [5]. To effectively manage, analyse and interpret such large amounts of data, powerful and efficient computational resources are essential. As a result, the development of bioinformatics platforms has become a priority for universities and research centres around the world [6]. This has led to an urgent need for efficient and scalable computational tools for data analysis. A cluster provides the necessary computational and storage resources to support bioinformatics analysis. In bioinformatics, a cluster, which is a group of interconnected computers, can be used to process large biological datasets efficiently and accurately. The use of clusters in universities and research centres has become crucial due to the ever-increasing amount of data generated by research activities. Several bioinformatics centres are being developed in Africa [5]. There are a growing number of initiatives that have established bioinformatics platforms on the African continent to support research that requires greater representativeness in Southern Africa [7]. However, the analysis of omics data is limited by both human and computational resources [5]. Bioinformatics in Africa currently lags behind other regions of the world, but several initiatives are helping to build capacity and knowledge in this important area. The Human Heredity and Health in Africa: H3Africa (https://h3africa.org/) initiative and H3AbioNet, the Pan African Bioinformatics Network: H3AbioNet (https://www.h3abionet.org), are working to integrate and communicate bioinformatics research across the continent. H3AbioNet focuses on building informatics infrastructure and human resources, supporting H3Africa projects and developing a data coordination centre. It also offers bioinformatics courses, both online and on-site in several African countries [6, 8–12].

In South Africa, bioinformatics has developed since the mid-1990s with the establishment of the South Africa National Bioinformatics Institute (SANBI). SANBI has expanded throughout the country, establishing computational biology units at universities and promoting undergraduate and postgraduate training in bioinformatics. Government involvement has also contributed to the growth of the field in South Africa, making it a leader in African bioinformatics [12–14].

In the early 2000s, west African countries, led by Nigeria and Ghana, began to introduce bioinformatics as an academic field through seminars, workshops and symposia [12, 15, 16]. The Nigerian Bioinformatics and Genomics Network was established in 2019 to meet the growing demand for bioinformatics experts and applications [12, 14–16].

Today, the field of bioinformatics is characterised by the constant growth of datasets, requiring ever-increasing computational power to tackle tasks such as genome assembly, gene expression analysis, protein structure prediction, phylogenetic inference, or drug design. The challenges of processing these large datasets are compounded by the diverse nature of bioinformatics tasks, which often require the use of different computational tools and algorithms. To address these challenges, BurkinaBioinfo (BBi) has planned to invest in high-performance computing (HPC) resources to meet the specific needs of bioinformatics research.

The aim of this paper is to report on the development and management of bioinformatics resources in Burkina Faso, as a process evaluation and as a comprehensive resource for anyone involved in capacity building in computational biology in low and middle income countries. We present an overview of the current state of bioinformatics research in Burkina Faso and highlight the importance of using a cluster for data analysis in universities and research centres, and for training and capacity building. We identify the key factors involved in setting up a bioinformatics cluster, including hardware and software requirements, network architecture and security measures. We provide comprehensive guidelines for configuring and optimising a bioinformatics cluster infrastructure, including the use of cluster management tools, job scheduling and parallel processing techniques. We also discuss the challenges of maintaining and upgrading a bioinformatics cluster, including data backup and recovery, system monitoring, and hardware maintenance, and suggest potential solutions to these problems. In addition, we highlight successful bioinformatics cluster case studies and examples from universities and research centres in Burkina Faso to demonstrate their impact on research outcomes and scientific discoveries.

## How did we pave the way for Burkina Faso's first bioinformatics platform?

At the beginning of the 1990s, the first life science research laboratories were established in universities and research centres in Burkina Faso. These laboratories were developed by the first national researchers to meet training needs and the country's development challenges. For example, laboratories with the capacity to perform ELISA tests and produce diagnostic antibodies enabled the first PhD students to be trained at the University of Ouagadougou in various fields of biological sciences.

This dynamic continued with some activities in genomics, often in collaboration with foreign universities and research centres. Researchers and university teachers were then trained in genomics in the laboratories of the French National Institute for Sustainable Development (IRD), the Centre for Research into Agricultural Development (CIRAD) and other university partners in Europe, the USA, Canada and Russia.

This dynamic has allowed the first laboratories to be equipped with PTC 100 generation thermal cyclers—PCR machine equipment from MJ Research, INC, PERKINS ELMER, etc.—for research and student training needs at the University of Ouagadougou and in the country's research centres. The capacity to work on DNA and RNA in the country really began in the late 1990s and became generalised in the 2010s. Technologies involving these molecules can now be carried out locally, although sequencing is still carried out in collaboration with advanced laboratories in Europe, the USA and Canada. Some of the research challenges in human health, plant and animal pathogens are thus being addressed in the laboratories.

The first sequencers have been purchased to equip research centres such as CIRDES: Centre International de recherche-développement sur l'élevage en zone subhumide (https://www.cirdes.org/), CERBA: Centre De Recherche Biomoleculaire Pietro Annigoni (https://cerba-burkina.org/) and hospitals in 2008-2012. These sequencers have started to generate sequencing data locally.

As genomics has developed in Burkina Faso, training in bioinformatics for handling the data generated has unfortunately not kept pace. The first bioinformatics courses were given at the University of Ouagadougou in 2005, as part of doctoral courses. They were essentially a presentation of the biological resources and tools for using them available on the web, such as NCBI. Young students were also introduced to phylogenetic analyses based on the Sanger sequencing data, using graphical software such as BIOEDIT, BLAST, DAMBE, or MEGA. Until 2015, there were no HPC resources dedicated to training, nor specialists in bioinformatics.

Starting in 2015, bioinformatics training courses was effectively launched at the University of Ouagadougou, in collaboration with IRD (Institut de Recherche pour le Développement), and more specially the SouthGreen bioinformatics platform in Montpellier, France. To meet the need of second-generation sequencing data analysis, the latter organized several training courses in Senegal and Burkina Faso in 2015 and 2016. These courses enabled west African researchers and students to acquire the skills needed to carry out metagenomic analyses, SNP polymorphism detection, or RNA-Seq differential expression. Analyses were carried out on a server using both a terminal and web applications such as galaxy. At the end of this first training cycle, in order to further develop skills in bioinformatics, the need emerged to set up a platform at the University of Ouagadougou, under the impetus of the genetics team in the biosciences laboratory of the UFR-SVT (Unité de Recherche et de Formation en Sciences de la Vie et de la Terre). The acquisition and installation of the computing servers was then carried out with the technical expertise of IRD bioinformatics team. The LMI Patho Bios (IRD-INERA) was the linchpin for the launch of the training courses at the University of Ouagadougou, which has since become Joseph KI-ZERBO University. Continuing to develop bioinformatics skills in depth and in a sustainable way, a young researcher was trained at the University of Paris (DUBii 2020 - 2022) with the support of the SouthGreen platform (IRD) and co-funding from the French Embassy in Ouagadougou and the Central and west African virus epidemiology: WAVE (https://wave-center.org/) program funded by the Bill and Melinda Gates Foundation and the Foreign, Commonwealth & Development Office.

Operational since 2019, the platform named BurkinaBioinfo: BBi (https://burkinabioinfo.github.io/) offers better training of students and professionals holding genomic data from different sequencing orders, as well as bioinformatics support for their research project. The platform draws upon the expertise of the Josep KI-ZERBO university: UJKZ (https://www.ujkz.bf/) and National Centre for Scientific and Technological Research: CNRST. It has made it possible to set up a better technical offer in terms of training in bioinformatics for Burkina Faso and the west African sub-region.

## How to increase genomics and bioinformatics expertise in Burkina Faso and the west African sub-region?

The BBi platform is a network of researchers from UJKZ and CNRST in Burkina Faso, and two international partners, H3ABioNet and i-Trop platform (IRD), with strong expertise in Bioinformatics and Genomics.

### Training of users

**Figure 1:**
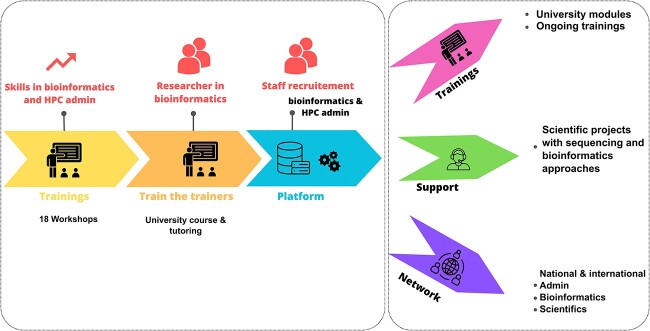
Diagram of the strategy for implementing training, establishing a bioinformatics platform in Burkina Faso, and creating a sub-regional network. The training targeted both biologists and computer scientists.

The training program in bioinformatics and IT administration was launched in 2019, targeting scientists and system administrators from several west African countries, including Benin, Burkina Faso, Côte d’Ivoire, Mali, Niger, Togo, and Senegal. The selected trainees comprised graduates, postgraduates, lecturers, and researchers with both theoretical knowledge and practical skills in molecular biology (Fig. 1).

**Figure 2:**
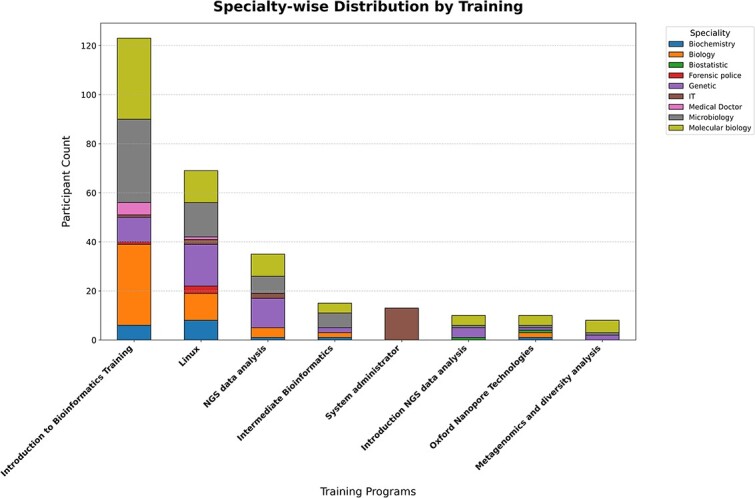
Specialty-wise distribution of participants across training programs. The figure illustrates the distribution of participants' specialties across various bioinformatics training programs. Each bar represents a training program, segmented by the participants' specialties, displayed as a stacked bar plot. The y-axis indicates the cumulative count of participants (from 2019 to 2023), while the x-axis lists the training programs, sorted by the total number of participants in descending order.

Between 2019 and 2023, a total of 18 training sessions were conducted, engaging approximately 284 participants (Fig. 2). The trainings covered a wide range of topics, including data production (Sanger sequencing, NGS, and Oxford Nanopore Technology), introductory bioinformatics, phylogenetic analysis, metagenomics, Oxford Nanopore Technologies (ONT) data analysis, and NGS data analysis (Fig. 2).

BBi's strategy focuses on broadening access to foundational training programs, such as Introduction to Linux and Introduction to Bioinformatics (IBT), to reach a wider audience. From these introductory courses, the best-performing participants are selected to advance to intermediate-level programs, such as Introduction to NGS Data Analysis and other intermediate bioinformatics topics. Finally, the top participants from the intermediate stage are chosen to enrol in advanced courses, including ONT Data Analysis and Metagenomics and Diversity Analysis (Fig. 2).

Through the H3ABioNet online classes, over 250 scientists got an additional capacity building in bioinformatics. These classes covered introduction to the field of bioinformatics, NGS data and 16S rRNA microbiome analysis. BBi platform hosted classrooms for seven H3ABioNet courses from 2021 to 2023 [10, 17].

The lesson modules offered have evolved in line with technological advances, including the recent addition of an ONT analysis module covering steps from sequencing to genome assembly. In the future, new modules will be introduced to address new technologies and evolving needs, such as single cell analysis.

The trainers are mainly biologists and bioinformaticians from Africa bioinformatics consortium supported by local trainers from research centre. To date, 105 user accounts have been created for access usage of the HPC. these users are coming from four countries in west Africa (Benin, Burkina Faso, Côte d’Ivoire, Senegal and Togo).

In addition to these bioinformatics training courses, the BBi platform, in collaboration with the i-Trop platform has developed training courses for system administrators of clusters (https://github.com/burkinabioinfo/burkinabioinfo.github.io). About three trainings were organized for west African admin engineers, in Burkina Faso and in France, covering a wide range of technologies and tools such. All training materials are available to all users under a CC BY licence d on the GitHub repository of the BBi platform (https://github.com/burkinabioinfo/burkinabioinfo.github.io).

The BBi platform has received financial and technical support from several programs or institutions, including IRD, WAVE, Biosciences Laboratory, Africa Wits-INDEPTH Partnership for Genomic Research (AWI-GEN), Strengthening expertise and bioinformatics to control antimicrobial (SEBA), and Centre de Formation, de Recherche et d’Expertise en sciences du Médicament (CEA-CFOREM).

## How to build the BBi platform?

Prior to the launch of this initiative, Burkina Faso faced a lack of bioinformatics infrastructure. This limitation forced researchers at the CNRST and UJKZ to juggle multiple responsibilities, including conducting research in genomics, biotechnology, and life and health sciences, while managing human resource training and securing research funding (Table 1).

**Table 1:** Mapping of laboratories producing genomic data in Burkina Faso.

Laboratories	Sequencing technologies	Type of sequencer	Locality
LCR	ONT llumina	MinION Miseq®	Ouagadougou
LNR-RAM	ONT	MinION	Bobo-Dioulasso
LNR FHV/AFROSCREEN	Illumina ONT	Miniseq® Iseq100® MinION	Ouagadougou
CNRFP	Sanger	SeqStudio	Ouagadougou
LNR Grippe	ONT	MinION	Ouagadougou
UGenoPath-OH/UJKZ	ONT	MinION	Ouagadougou
IRSS-Nanoro	Illumina ONT	Miseq MinION	Nanoro
LaReBio	Iron Torrent	Ion torrent S5 Prime + Ion	Ouagadougou
LVBV/INERA	ONT	MinION	Ouagadougou
LaBioSA	Sanger	SeqStudio	Ouagadougou
LNR-FHV	Illumina	Iseq100	
LNR- VIH	Sanger	SeqStudio	Ouagadougou
LNR-HPV	Sanger Iron Torrent	SeqStudio Ion torrent	Ouagadougou
LR-MIP	ONT	MinION	Bobo-Dioulasso
CRSN	Sanger Iron Torrent	Ion torrent S5 Prime + Ion	Nouna
GRAS	ONT	MinION	Ouagadougou
LABIOGENE/UJKZ	ONT	MinION	Ouagadougou

### First step: identifying the needs

The first step in setting up a bioinformatics cluster was to identify the specific needs of the researchers in Burkina Faso. This step involved working with the researchers to understand their data processing needs, storage capacity, and computational requirements. Based on the requirements, the specifications of the cluster were designed to meet the specific needs of the researchers.

At the time cluster was conceived, the type of genomic data generated or hosted by different research teams ranged from genotyping (PCR and array) to whole genome sequences data (Table 1).

### Second step: selecting appropriate hardware

Once the researchers' needs had been identified, the next step was to select the appropriate hardware for the cluster. This involved selecting processors, storage devices and networking equipment capable of handling the large volumes of data generated by the research projects. The availability of a data centre within the UJKZ made this process much easier. The hardware was specifically designed to be scalable, allowing for future expansion as the research needs grow. It was also chosen to ensure compatibility with the software and bioinformatics tools to be installed. The architecture was carefully planned to facilitate maintenance and ensure long-term reliability and efficiency.

### Third step: installing software and specific tools

The next essential step was the installation and configuration of software and bioinformatics tools. This process included setting up operating systems, database management systems, and specialized bioinformatics applications. The installation was carried out in collaboration with the system administrator responsible for i-Trop and rigorously tested to ensure stability and optimal performance.

## Challenges and prospects of the platform

The organization of a bioinformatics platform in Sub-Saharan Africa in general and in Burkina Faso presents many challenges, but also offers great opportunities for scientific research and economic development in the country.

The challenge in setting up and running an efficient bioinformatics platform include.

- One of the challenges related to the lack of qualified personnel, trained in bioinformatics and capable of running, maintaining, and monitoring the platform's computer tools. Indeed, Bioinformatics is a complex discipline that requires skills in computer science, biology, and statistics. The lack of qualified personnel in these fields can make it difficult to set up and manage a bioinformatics platform. There was a need for a critical mass of trained bioinformaticians. In the specific case of our BBi platform, we contribute to address this challenge by training a specialist in Bioinformatics. In fact, when BBi was launched, we had only two proficient bioinformaticians and one system administrator. Over the years, through a mentoring system, we have gradually increased the critical mass of proficient bioinformaticians and trainers by identifying and uplifting the best trainees. In order to ensure the efficiency of the platform, the capacity building must continue with a pyramid shaped training system in mind, ensuring sustainability. Thus, with this capacity building model in mind, the BBi Platform has undertaken since 2018, a series of training courses for professionals and students to provide them with introductory knowledge in bioinformatics. Thus, in collaboration with H3ABioNet, and with the colleagues of LMI Patho Bios, a series of certification trainings were organized for capacity building. These trainings are essential to create a community of ``bioinformaticians'' with a bioinformatics spirit in an international scientific context marked by the development of bioinformatics tools.
- The second major challenge pertains to the enhancement of the platform's capacity with instruments and computer and electronic equipment capable of ensuring the platform's proper and efficient operation as this is the case of the BBi platform. Maintaining the safety of the platform, and increasing processing capacity, developing automated workflows remains a priority. Stable internet and electricity infrastructure in Sub-Saharan Africa in general and specifically in Burkina Faso is limited, which can make it difficult for users to always access the platform remotely. In other to overcome challenges with power supplied, BBi purchased a dedicated generator has backup in case off load shedding, we are planning as well a solar plant for sustainability. In addition to infrastructure limitations as a challenge, there is a need to consider the administrative and financial constraints about procurement system in Burkina Faso. Indeed, the public procurement system in Burkina Faso, although nationally accepted, slows down the acquisition process or increases at times the acquisition costs of certain equipment whose approved suppliers are based outside the country and sometimes without any inland relay. This is a challenge that could be overcome by having a good interaction with the institutional management.

However, the establishment of a bioinformatics platform in Sub-Saharan Africa and in Burkina Faso, offers great opportunities for scientific research and economic development in the country region. These prospects include:

1. Scientific research: A bioinformatics platform can help researchers analyse genomic data and promote health research, new crop varieties, and environmental solutions. Bioinformatics can help solve many health, agricultural, and environmental problems in the Sub-Saharan Africa including Burkina Faso.
2. Economic development: Bioinformatics can help stimulate economic development in Sub-Saharan Africa by creating new jobs in scientific research, computer science, and data management. A bioinformatics platform can also help companies develop new products and services based on genomic data.

Despite significant challenges in establishing and maintaining HPC infrastructures, Africa has witnessed notable success stories, driven by innovative approaches to data management and collaboration. At the pan-African level, initiatives such as the African Open Science Platform: AOSP (https://aosp.org.za/) have spearheaded the creation of repositories and frameworks that promote open science, enabling researchers to store, share, and access datasets in key areas like public health, agriculture, and climate science.

National efforts further exemplify progress, with countries like South Africa and Kenya leading the way. In the bioinformatics field, South African National Bioinformatics Institute: SANBI (https://www.sanbi.ac.za/) provides open access to datasets, facilitating research across the continent, while Kenya’s National Data Center serves as a central hub for data management and sharing. Discipline-specific repositories have also emerged, such as the Human Heredity and Health in Africa (H3Africa) project, which supports genomics research, and CGIAR’s Open Data Initiative, focused on advancing agricultural research. In North Africa, Morocco has set up the BIOINFORMATICS LAB through University Mohammed VI Polytechnic (UM6P).

South Africa stands out as a regional leader, hosting more than half of the continent's bioinformatics infrastructure. Through strong collaborations among universities, research centres, and policymakers, the country has become a global leader in bioinformatics.

Compared to other HPC platforms in Africa, which are primarily dedicated to high-performance computing in fields such as mathematics, physics, and meteorology, BBi stands out as being exclusively focused on bioinformatics and computational biology. However, efforts are underway to expand the platform's accessibility to other disciplines requiring high-performance computing, ensuring that a broader range of scientific fields can benefit from its capabilities.

Platforms like BBi aim to draw inspiration from such initiatives, combining academic innovation with robust national policies to promote training, job creation, and the development of endogenous solutions to Africa’s pressing challenges in health, agriculture, and economic development. As part of its future plans, BBi aims to establish collaborations with other bioinformatics platforms both within Africa and word. The initiative will leverage opportunities provided by organizations such as the Research Data Alliance: RDA (https://www.rd-alliance.org/), CODATA (https://codata.org/)and more, which support data sharing and open science initiatives. Additionally, BBi plans to engage with African scientific societies in bioinformatics, such as the African Society for Bioinformatics and Computational Biology (ASBCB), to foster stronger connections within the continent's research community and advance bioinformatics capacity in the region.

## Conclusion

The establishment of BBi platform marks a pivotal advancement in the development of bioinformatics infrastructure in Burkina Faso. By addressing the specific needs of researchers at CNRST and UJKZ, the initiative has not only deployed suitable hardware but also provided essential training for effectively utilizing the cluster. Leveraging the experiences of other African countries, BBi has designed targeted training programs for students and young researchers, equipping them with foundational bioinformatics knowledge, programming expertise, and technical skills.

While challenges such as high bandwidth costs and limited international network connectivity persist, the UJKZ bioinformatics platform is steadily building the infrastructure necessary to support research and fostering a growing interest in bioinformatics within Burkina Faso's academic and scientific communities. Importantly, this initiative paves the way for expanding genomic research and harnessing cutting-edge technologies like next-generation sequencing. We hope that our efforts will inspire and guide similar initiatives in other countries, contributing to the broader development of bioinformatics across the region.

BBi platform, like other platforms in Africa, places a strong emphasis on bioinformatics and computational biological sciences, aiming to strengthen local capacities and foster innovation in these critical fields.

### Perspectives

As part of its development, BBi plans to strengthen collaborations with other platforms in Europe, the United States, and Asia to promote scientific and technical exchanges. Additionally, to facilitate access to bioinformatics analyses and extend access to wider community, we aim to develop internet-based access through applications such as Jupyter book and Galaxy, enabling researchers less familiar with programming to conduct their analyses effectively.

Based on user needs, BBi will also develop customized web tools to support their work and address the specific requirements of their research.

**Key Points**

- Bioinformatic plays a significant role in genomics, proteomics, metabolomics, agriculture, and environmental conservation research. This highlights the multifaceted applications of bioinformatics and its necessity for scientific advancements in west Africa.
- Establishing bioinformatics platforms with HPC resources is essential for universities and research centres in west Africa.
- The establishment of the BBi platform in 2019 marked a major milestone, providing advanced training and bioinformatics support for research projects, leveraging the expertise of Joseph KI-ZERBO University and the National Centre for Scientific and Technological Research (CNRST).
- Collaboration has facilitated the development and delivery of high-quality training programs. Financial and technical support from various programs and institutions, including IRD, WAVE, SEBA, AWI-GEN, and CEA-CFOREM, has been crucial in sustaining and expanding these initiatives. This robust support network ensures the continuous development of genomics and bioinformatics expertise in Burkina Faso and the wider west African sub-region.

## Data Availability

All data supporting the findings of this study are fully accessible. The datasets generated and analyzed during this study are available through the links provided in the manuscript.
